# Kawasaki disease in Kenya and review of the African literature

**DOI:** 10.1186/s12969-024-00977-1

**Published:** 2024-04-14

**Authors:** A. Migowa, CM. Njeru, E. Were, T. Ngwiri, I. Colmegna, C. Hitchon, R. Scuccimarri

**Affiliations:** 1 Department of Pediatrics and Child Health, Aga Khan University Medical College (East Africa), Nairobi, Kenya; 2 Department of Pediatrics, Gertrude’s Children’s Hospital, Nairobi, Kenya; 3 Division of Rheumatology, Department of Medicine, McGill University Health Centre, Montreal, QC Canada; 4 Section of Rheumatology, Department of Internal Medicine, University of Manitoba, Winnipeg, Manitoba, Canada; 5 Division of Pediatric Rheumatology, Department of Pediatrics, McGill University Health Centre, 1001 boul. Décarie, A04.6306, H4A 3J1 Montreal, QC Canada

**Keywords:** Kawasaki Disease, Vasculitis, Pediatric Rheumatology, Africa, Kenya, Global Health

## Abstract

**Background:**

Kawasaki disease has been described across the globe, although publications from Africa are limited. To our knowledge, there are no publications on Kawasaki disease from Kenya, which triggered this report.

**Methods:**

A retrospective cross-sectional study was undertaken to identify in-patients with a discharge diagnosis of Kawasaki disease, over 2 different 5-year periods, at two pediatric hospitals in Nairobi, Kenya. We reviewed the medical records of all patients and report their clinical findings, diagnostic workup and treatment. In addition, we undertook a detailed review of the literature.

**Results:**

Twenty-three patients with Kawasaki disease were identified, of those 12 (52.2%) had incomplete disease. The mean age was 2.3 years (SD+/-2.2) (range 0.3–10.3) with a male to female ratio of 1:1. The mean duration of fever at diagnosis was 8.3 days (SD+/-4.7) (range 2–20). Oral changes were the most common clinical feature and conjunctivitis the least common. Thrombocytosis at diagnosis was seen in 52% (12/23). Twenty-one patients (91.3%) were treated with intravenous immunoglobulin and all except 1 received aspirin. Baseline echocardiograms were performed in 95.7% (22/23) and found to be abnormal in 3 (13.6%). Follow-up data was limited. Our literature review identified 79 publications with documented cases of Kawasaki disease in children from 22 countries across the African continent with a total of 1115 patients including those from this report. Only 153 reported cases, or 13.7%, are from sub-Saharan Africa.

**Conclusions:**

This is the first publication on Kawasaki disease from Kenya and one of the largest reports from sub-Saharan Africa. It is the first to have a complete review of the number of published cases from the African continent. Challenges in the diagnosis and management of Kawasaki disease in many African countries include disease awareness, infectious confounders, access and cost of intravenous immunoglobulin, access to pediatric echocardiography and follow-up. Increasing awareness and health care resources are important for improving outcomes of Kawasaki disease in Africa.

**Supplementary Information:**

The online version contains supplementary material available at 10.1186/s12969-024-00977-1.

## Background

Kawasaki disease (KD) is an acute, self-limited vasculitis of unknown etiology that affects predominantly infants and young children [1]. It was first described by Dr. Tomisaku Kawasaki in Japan in 1967 [2]. The diagnosis of KD is clinical. It is based on the presence of fever lasting ≥ 5 days, together with at least four of the five following physical findings in the absence of an alternative explanation: bilateral non-exudative conjunctivitis, oral mucous membrane changes (i.e., erythema and cracking of the lips, injected pharynx, and/or strawberry tongue), polymorphous rash, cervical lymphadenopathy (at least one lymph node ≥ 1.5 cm in diameter; usually unilateral) and peripheral extremity changes [i.e., erythema and edema of the hands and feet (acute phase); and/or periungual desquamation (subacute phase)] [1].

Coronary artery aneurysms (CAA) develop in approximately 25% of untreated children with KD, which can lead to myocardial infarction, ischemic heart disease or sudden death [1]. A normal baseline echocardiogram does not exclude the possibility of the development of CAA; thus, the American Heart Association guidelines suggest echocardiograms at baseline and repeated at 1 to 2 weeks and 4 to 6 weeks following treatment [1]. Combined treatment with high dose intravenous immunoglobulin (IVIG) (2 g/kg) and aspirin decreases the incidence of CAA to < 5% [1].

Timely diagnosis and adequate treatment are imperative in KD. However, KD’s diagnosis can be challenging since its signs and symptoms are non-specific and often similar to other common pediatric infectious illnesses; symptoms can evolve over the first week or be evanescent; and patients can have incomplete presentations [3]. Even patients with incomplete KD can develop CAA [1]. Given that KD remains a clinical diagnosis with no diagnostic test available, meticulous history-taking and thorough physical examination are essential [4]. The assessment of the clinical criteria must include the caregivers’ descriptions together with health professionals’ direct observations [4]. This diagnosis should also be considered in children with prolonged unexplained fever, even if there are no or few clinical features of KD [1]. Unique clinical findings of KD include inflammation at the site of Bacillus Calmette–Guérin vaccination [4–6], which may occur in up to 50% of vaccinated patients [5]; and periungual desquamation, which usually occurs 2 to 3 weeks after the onset of fever [1].

KD is the most common cause of acquired heart disease in children in Japan, North America and Europe [7, 8]. In low and middle-income countries, rheumatic fever remains the main cause of acquired heart disease [9], however KD is not far behind [5, 7, 8]. In Africa, the true incidence of KD is unknown. Nonetheless, Takayasu’s arteritis and KD are the most commonly reported vasculitides [10].

In 1979, Elamin reported the first two cases of KD from the African continent from Zambia [11]. Subsequently there were many case series and reports of KD in children from Africa but, to our knowledge, there are no publications on KD from Kenya. Herein, we describe a case series of 23 children diagnosed with KD, over 2 different 5-year periods, at two hospitals in Nairobi, Kenya.

## Methods

A retrospective study was undertaken to identify in-patients with a discharge diagnosis of KD from either Gertrude’s Children’s Hospital (GCH) or Aga Khan University Hospital Nairobi (AKUHN). Both institutions are private not-for-profit hospitals located in Nairobi, the capital of Kenya, and have electronic medical records (EMR) since 2011 at GCH and 2013 at AKUHN.

Patients with a discharge diagnosis of KD according to the International Classification of Disease 10th edition code (ICD-10) between January 1st 2011 and September 30th 2015 at GCH, and January 1st 2013 to December 31st 2017 at AKUHN were identified through EMR. Medical records were reviewed and data on history, clinical examination, investigations and treatment were extracted. Four cases reported here were previously included in a study published by our group describing the spectrum of in-patient pediatric rheumatic diagnoses at GCH in 2011 [12]. We report descriptive statistics and compared groups using Student T tests for continuous variables, after confirming acceptable Kurtosis and skewedness, and Chi-square tests for categorical variables.

A detailed review of the literature was undertaken in PubMed® and Google Scholar® using combinations of the terms Kawasaki Disease, Kawasaki Syndrome, Mucocutaneous Lymph Node Syndrome and Africa. In addition, the term Africa was replaced by each of the 54 African countries independently. All full articles were included without language restrictions. Adult cases, and those related to Infantile Polyarteritis Nodosa, or Multisystem Inflammatory Syndrome in Children from COVID-19, were excluded.

## Results

Twenty-three cases of KD were identified: 8 at GCH and 15 at AKUHN (see Table 1). The sex distribution was similar (12 boys: 52.2% and 11 girls: 47.8%). The mean age of patients was 2.3 years (SD+/-2.2) (range 0.3–10.3). Twelve patients (52.2%) had incomplete KD. The frequency of incomplete KD was similar at GCH and AKUHN (62.5% vs. 46.7; *p* = 0.47). The mean duration of fever at diagnosis was 8.3 days (SD+/-4.7) (range 2–20). There was a trend towards longer duration of fever in those with incomplete KD (10.08 vs. 6.45; *p* = 0.06). There was also a trend towards a longer duration of fever in patients at GCH compared to AKUHN (11.3 vs. 6.8; *p* = 0.07). All patients had oral changes, 87% (20/23) had skin rashes, 78% (18/23) had extremity changes, 52% (12/23) had unilateral cervical lymphadenopathy, and 48% had (11/23) non-exudative conjunctivitis. In both groups, oral changes, skin rashes and extremity changes were the most common clinical features of KD.

**Table 1:** Description of the Kenyan Kawasaki disease cases from AKUHN and GCH

	AKUHN	GCH	TOTAL
	*n* (%)		
**No. of cases**	15 (65)	8 (35)	23 (100)
**Age [mean+/-SD, range, years]**	2+/-1.5 [0.3–5.7]	3+/-3.2 [0.7–10.3]	2.3+/-2.2 [0.3–10.3]
**Sex - Males**	8 (53.3)	4 (50)	12 (52.2)
**Duration of fever at diagnosis [mean+/-SD, range, days]**	6.8+/-3.4 [2–13]	11.3+/-5.3 [5–20]	8.3+/-4.7 [2–20]
**Incomplete KD**	7 (46.7)	5 (62.5)	12 (52.2)
**Clinical Features**			
**Fever**	15 (100)	8 (100)	23 (100)
**Oral Changes**	15 (100)	8 (100)	22 (95.7)
**Rash**	13 (86.7)	7 (87.5)	20 (87)
**Extremity changes**	12 (80)	6 (75)	18 (78.3)
**Cervical lymphadenopathy**	9 (60)	3 (37.5)	12 (52.2)
**Nonexudative conjunctivitis**	8 (53.3)	3 (37.5)	11 (47.8)
**Laboratory Investigations**			
**CRP (> 5 mg/ L)**	12/12 (100)	8/8 (100)	20/20 (100)
**Erythrocyte Sedimentation** **Rate (> 15 mm/hr)**	8/9 (88.9)	5/5 (100)	13/14 (92.9)
**Platelets (≥** 450*10^**9**^**)**	8/15 (53.3)	4/8 (50)	12 (52.2)
**Treatment**			
**IVIG**	14 (93.3)	7 (87.5)	21 (91.3)
**Aspirin**	14 (93.3)	8 (100)	22 (95.7)
**Corticosteroids**	2 (13.3)	2 (25)	4 (17.4)
**Echocardiogram**			
**Number of Baseline** **Echocardiograms**	14 (93.3)	8 (100)	22 (95.7)
**Number of Baseline** **Echocardiograms with** **CAA**	1/14 (7.1)	1/8 (12.5)	2/22 (9.1)

In those whose inflammatory markers were measured (*n* = 20), all had elevated C-reactive protein (CRP). Thrombocytosis at diagnosis was seen in 52% (12/23). Twenty-one of the 23 patients (91.3%) were treated with IVIG, and all patients except 1 received aspirin. In addition, 4 patients (17.4%) were treated with corticosteroids: one for myocarditis; one for failed response to IVIG; another for management of a suspected allergic reaction; while in another the clinical context in which steroids were used was unclear. Baseline echocardiograms were performed in 95.7% (22/23) and found to be abnormal in 3 (13.6%): 2 patients had CAA (1 from each hospital); and another had myocarditis. Only 6/23 (26.1%) patients were documented to have a follow-up echocardiogram. Since a normal baseline echocardiogram does not exclude the possibility of later development of coronary aneurysms, the true CAA incidence among the KD patients in this report could not be established.

Our literature review identified 79 publications with documented cases of KD in children from 22 countries across the African continent with a total of 1115 patients including those from this report (see Table 2) [6, 11–88]. The first cases of KD were described from Zambia [11]. Subsequently there were reports of children with KD from South Africa, Ivory Coast, Tunisia, Senegal, Nigeria, Sudan, Democratic Republic of the Congo, Madagascar, Ghana, Algeria, Morocco, Togo, Cameroon, Egypt, Tanzania, Uganda, Liberia, Gabon, Guinea and Libya [6, 13–88]. Only 153 reported cases, or 13.7%, are from sub-Saharan Africa.

**Table 2:** Review of reported cases of Kawasaki disease in Africa

Country	Number of Cases per Report	Total Cases per Country	Reference Numbers
Algeria	133, 1**, [64]*, 62	196	47, 48, 49, 85
Cameroon	1, 1	2	51, 80
Democratic Republic of the Congo	2, 11	13	26, 43
Egypt	2**, 4, 64, 22**	92	55, 56, 57, 60
Gabon	33	33	81
Ghana	3	3	36
Guinea	1	1	87
Ivory Coast	1	1	15
Kenya	23, [4]*	23	Current paper, 12
Liberia	1	1	78
Libya	71	71	88
Madagascar	1, 1	2	30, 35
Morocco	23, 1, 1, 1, 1, 1, 359^#^	387	49, 63, 69, 72, 75, 83, 86
Nigeria	[1]*, 1, 1, 1, [1]*, 1, 2, 1, [1]*, 8, [2]*, 1, 1, [2]*, 1	18	6, 21, 34, 39, 40, 41, 44, 45, 52, 53, 54, 70, 76, 77, 79
Senegal	1, 1	2	17, 68
South Africa	2, 4, [6]*, 12, 1, [1]*, 4**, 1, 1, 19	44	13, 14, 18, 19, 20, 22, 23, 25, 31, 46
Sudan	2, 2	4	24, 71
Tanzania	2, 2, 1	5	59, 67, 73
Togo	1, 1	2	50, 82
Tunisia	2, [1]*, 7**, 14**, 29**, 1**, 1**, [1]*, [1]*, 31, 1, 33, 25**, 1, 1, 1, 65	212	16, 27, 28, 29, 32, 33, 37, 38, 42, 49, 58, 61, 62, 64, 65, 74, 84
Uganda	1	1	66
Zambia	2	2	11
Total cases from North Africa		962	
Total cases from sub-Saharan Africa		153	
**Total cases**		**1115**	

*Duplicate cases; **Some of these cases may have been included in other papers, but given uncertainty, they were counted as separate cases; ^#^Removed COVID-19 related cases [379 − 20 (42% of 47)]

## Discussion

Over two different 5-year periods, 8 cases of KD were identified at GCH and 15 cases from AKUHN. To our knowledge, these are the first KD cases to be reported from Kenya. In both cohorts, oral changes were the most common clinical feature and conjunctivitis was the least common. Conjunctivitis was less common in this population as compared to reports from North America [3]. As expected, CRP was elevated in all patients in whom it was tested. More than 50% had thrombocytosis at diagnosis possibly reflecting the longer duration of fever prior to diagnosis. Thrombocytosis may be a helpful laboratory finding to aid with diagnosis, in patients presenting with 7 days of fever or more, given that it is often present at this point in the disease course [1]. There were proportionally more incomplete KD presentations at GCH as compared to AKUHN, although this finding was not statistically significant. It appears that patients who presented to GCH had a longer fever duration, with the fever often persisting even after other clinical features had already resolved. Delayed presentations will often have more limited clinical features, which can make diagnosing KD more challenging. Given that the GCH cases were identified in the earlier medical record review, it is possible that reduced awareness of KD in Kenya at that time led to longer symptom duration prior to diagnosis.

Our literature review identified cases of KD in children from 22 countries across the African continent with a total of 1115 patients including those from this report [6, 11–88]. The largest KD cohorts are from North Africa [47, 57, 84, 86, 88] with > 350 KD patients in the largest report [86]. The reports from North Africa make up 86.3% of the published African cases. The largest reports, outside of North Africa, include 8 cases (1987–1988) from Johannesburg, South Africa [19], 8 (2011–2016) from Lagos, Nigeria [53], 11 (2003–2014) from Brazzaville, Democratic Republic of the Congo [43] and 33 (2014–2021) from Libreville, Gabon [81]. The case series from Gabon included 2 years from the COVID-19 pandemic and it is unclear how many of the cases presented were in that 2-year period and whether they had been exposed to COVID-19 [81]. Our report is one of the largest in sub-Saharan Africa.

Even though North Africa reported the largest number of cases, KD is still under-recognized and underdiagnosed in this region. Among children from the North African countries living in Quebec, Canada, the incidence of KD was 4 to 12 times higher than that reported in the countries of origin [49]. Countries in Africa can be compared to India, where KD is still not frequently diagnosed, and mortality is higher than in high-income countries [7]. Educational campaigns targeting both health care professionals and the lay public may increase KD awareness and contribute to earlier diagnosis [7] as was seen during the COVID-19 pandemic with Multisystem Inflammatory Syndrome in Children.

In our KD cases, the mean fever duration was 8.3 days (SD+/-4.7) (range 2–20). Mabiala and co-workers, in their case series from Democratic Republic of the Congo, also found that patients had prolonged fever on admission (mean 12.8 days; range 6–30) [43]. In a large series from Algeria of 133 patients, Boudiaf et al. proposed that the long duration of fever (13 ± 6 days) related to a marked delay in diagnosis of KD from the referral centers to their public tertiary care hospital [47]. They postulated that most delays were likely due to the difficulty in distinguishing KD from bacterial and viral infections [47], which was also likely the case in our cohort. In Nigeria, Animasahun et al. highlighted that two-thirds of patients in their series had been diagnosed as having other conditions and had been administered antimalarials and antibiotics [53]. They state that KD is only rarely considered as the likely diagnosis on initial evaluation [53].

Mabiala and colleagues highlighted challenges in diagnosing incomplete KD due to the lack of availability of echocardiography at their centre and the financial constraints of the population to access this resource [43]. When abnormal, echocardiography can be a useful adjunct to diagnosis especially in cases of incomplete KD [4]. In our case series, most patients (95.7%) had baseline echocardiograms. However, it is unclear how many underwent follow-up echocardiograms as this information was only documented for 6 patients. Unfortunately, follow-up echocardiogram results were also often lacking in many of the African reports with outcomes unknown beyond the acute illness.

Another challenge in resource-limited countries is access to trained pediatric cardiologists [8]. Echocardiography is operator-dependent and requires expertise, especially in young children [8]. Echocardiograms are often done by adult cardiologists, or by technologists, who may not have the expertise and training to assess the coronary arteries in an uncooperative infant or child [8]. As such, the echocardiography reports are often incomplete and inaccurate [8]. In addition, assessment of coronary arteries with calculation based on body surface area (Z-scores) may not be done routinely [8].

The goal of treatment is to control inflammation as rapidly as possible, preferably within 10 days of illness onset. IVIG is the treatment of choice [1]. Unfortunately, the cost of IVIG is prohibitive [5, 8, 53, 89]. This makes this treatment inaccessible to most patients in resource-limited countries, especially if caregivers must pay for this expense themselves [5, 8]. Ultimately, access and availability of resources is a key determinant to the treatment and management of KD patients in Africa. In our series, 91.3% of patients received IVIG, and all but one received aspirin. Given that these patients were seen at private hospitals, it is possible that this population had more financial resources to access IVIG compared to the general population of Kenya and other sub-Saharan countries. Similarly, the majority of patients from the large North African reports received IVIG and aspirin [57, 84, 86, 88]. However, in the case series from Ghana, Democratic Republic of the Congo and Gabon, patients only received aspirin [36, 43, 81] as was the case in many of the other reports from sub-Saharan Africa. In the Congolese case series, although the treating physicians recognized that IVIG was the treatment of choice, it was not accessible or it was deemed that patients presented too late for IVIG to be effective [43]. A case report from Madagascar (from 2008) and another from Cameroon (from 2022) described that IVIG was not available locally, and had to be imported from France, delaying this treatment [30, 80]. A case series, in 2017 from Nigeria, reported that access to IVIG in their country was limited, and when available, it could only be accessed at one hospital [53]. However, access to IVIG may have improved in some African countries. In 2022, Sokunbi et al., from Nigeria, reported that 60.7% of those treated for Multisystem Inflammatory Syndrome in Children were able to receive IVIG [89]. However, these authors state that IVIG was administered only to patients whose caregivers could access and pay for it given that it is very expensive and not readily available [89]. This highlights the ongoing challenges to accessing this important treatment for KD in many African countries.

Corticosteroids are the treatment of choice for most vasculitides and would be considered the most economical alternative to IVIG in resource-limited settings [5]. However, the American Heart Association currently only recommends the use of corticosteroids as rescue therapy in IVIG-resistant patients; and concomitantly, with IVIG and ASA at diagnosis, if the patient can be identified at high risk for IVIG resistance [1]. Even though there is no data to support the use of corticosteroids as the primary treatment of KD, corticosteroids may be the best current alternative in resource-limited settings when IVIG is not available or not affordable [5]. Given inaccessibility of IVIG, corticosteroids was used to treat KD in a few of the African reports [30, 51, 78, 82]. However, the major concern with this approach would be using corticosteroids in the context of an undiagnosed potentially life-threatening infection [5]. It has been suggested that a pragmatic approach would be careful observation and possibly empiric antibiotic treatment for commonly suspected pathogens during the initial steroid treatment, particularly if diseases such as typhoid cannot be excluded [5]. An evidence-based approach for the appropriate use of affordable alternatives to IVIG is needed [5].

## Conclusions

The diagnosis and management of KD in resource-limited countries of Africa is challenging. The polymorphic nature of KD, including incomplete presentations, the similarity of the clinical features with common infectious illnesses, and the potential of resolution of clinical features at time of assessment, needs to be recognized. Improving the care of children with KD will require increasing awareness among healthcare professionals about the complications associated with the lack of recognition/treatment of this disease, enhancing access to echocardiograms and specialists to interpret them (i.e., pediatric cardiologists), facilitating access to subsidized IVIG, and ensuring follow-up with echocardiograms. Studies evaluating the role of corticosteroids when IVIG is not accessible needs to be carefully considered due to the risk of infectious confounders.

### Electronic supplementary material

Below is the link to the electronic supplementary material.

Supplementary Material 1

## Data Availability

The datasets generated and/or analysed during the current study are not publicly available but are available from the corresponding author on reasonable request.

## Abbreviations

KD: Kawasaki Disease
SD: Standard Deviation
CAA: Coronary artery aneurysms
IVIG: Intravenous Immunoglobulin
GCH: Gertrude’s Children’s Hospital
AKUHN: Aga Khan University Hospital Nairobi
ICD-10: International Classification of Disease 10th edition code
COVID-19: Coronavirus Disease 2019
CRP: C-Reactive Protein
