# Supplementary material for: Kawasaki disease in Kenya and review of the African literature
Source: Pediatr Rheumatol Online J. 2024 Apr 14;22:43. doi: 10.1186/s12969-024-00977-1 (PMC11016229; doi:10.1186/s12969-024-00977-1)
Supplement: Supplementary file 1 — Supplementary Material 1 [file 12969_2024_977_MOESM1_ESM.pdf]

Professors Angelo Ravelli and Tadej Avčin
Dr Wafa Hamdi
Editors and Guest Editor
Pediatric Rheumatology

January 30, 2024

Dear Editorial team,

We are submitting our manuscript entitled "Kawasaki Disease in Kenya and Review of the African Literature" as an original research article to *Pediatric Rheumatology* for the collection "Context Specific Paediatric Rheumatology Care-Lessons from Africa". We describe a case series of 23 patients with Kawasaki Disease from 2 pediatric centres in Nairobi, Kenya. In addition, we performed a literature review and identified 79 publications on Kawasaki Disease in children from 22 countries across the African continent with a total of 1115 patients (including the patients from Kenya).

The senior authors on this paper are Canadian researchers who procured funding to study pediatric rheumatic diseases in Kenya in 2013-2015. There were no pediatric rheumatologists in Kenya during that period. This research enabled Canadian/African collaborations that led to the training of Dr. Angela Migowa, one of the first pediatric rheumatologists in East Africa. When she returned to Kenya, after her fellowship at McGill University, she was able to use similar methods to those we had previously used at Gertrude's Children's Hospital, at Aga Khan University Hospital, to get a more complete description of Kawasaki Disease in Nairobi.

We believe this manuscript is an important contribution to the field as it would be the first publication on Kawasaki Disease from Kenya and one of the largest reports from sub-Saharan Africa. In addition, the literature review we performed would give your readers a global perspective of Kawasaki Disease in Africa including its unique challenges in diagnosis and management. It would also give previous published literature from Africa, on Kawasaki Disease, an opportunity to be highlighted. It meets the theme of "Paediatric Rheumatology Care-Lessons from Africa".

All authors have approved the manuscript for submission and do not have any competing interests. The content of this manuscript has not been published or submitted for publication elsewhere.

Thank you for considering our manuscript.

Yours sincerely,

Rosie Scuccimarri, MD
Pediatric Rheumatologist, McGill University Health Centre
Associate Professor, Department of Pediatrics, McGill University
